# A dual inhibitor targeting HMG-CoA reductase and histone deacetylase mitigates neurite degeneration in *LRRK2-G2019S* parkinsonism

**DOI:** 10.18632/aging.104165

**Published:** 2020-11-24

**Authors:** Chin-Hsien Lin, Han-Yi Lin, Jim-Min Fang, Ching-Chow Chen

**Affiliations:** 1 Department of Neurology, National Taiwan University Hospital, College of Medicine, National Taiwan University, Taipei 100, Taiwan; 2 Department of Chemistry, National Taiwan University, Taipei 106, Taiwan; 3 Department of Pharmacology, College of Medicine, National Taiwan University, Taipei 100, Taiwan

**Keywords:** Parkinson’s disease, HMG-CoA reductase, histone deacetylase, leucine-rich repeat kinase, tubulin

## Abstract

Parkinson’s disease (PD) is among the most common neurodegenerative disorders, and its etiology involves both genetic and environmental factors. The leucine-rich repeat kinase (LRRK2) G2019S mutation is the most common genetic cause of familial and sporadic PD. Current treatment is limited to dopaminergic supplementation, as no disease-modifying therapy is available yet. Recent evidence reveals that HMG-CoA reductase (HMGR) inhibitors (statins) exert neuroprotection through anti-neuroinflammatory effects, and histone deacetylase (HDAC) inhibitors mitigate neurodegeneration by promoting the transcription of neuronal survival factors. We designed and synthesized a dual inhibitor, statin hydroxamate JMF3086, that simultaneously inhibits HMGR and HDAC, and examined its neuroprotective effects on *LRRK2-G2019S* parkinsonism. JMF3086 restored dopaminergic neuron loss in aged *LRRK2-G2019S* flies and rescued neurite degeneration in primary hippocampal and dopaminergic neurons isolated from transgenic *LRRK2-G2019S* mice. The molecular mechanisms included downregulation of ERK1/2 phosphorylation, increased anti-apoptotic Akt phosphorylation, and inhibition of GSK3β activity to maintain cytoskeletal stability in stably transfected *LRRK2-G2019S* SH-SY5Y human dopaminergic cells. JMF3086 also promoted a-tubulin acetylation and kinesin-1 expression, facilitating antegrade mitochondrial transport in axons. Our findings demonstrate that JMF3086 exerted beneficial effects on restoring *LRRK2-G2019S* neurite degeneration by maintaining microtubule stability. This dual-target compound may be a promising mechanism-based therapy for PD.

## INTRODUCTION

Parkinson’s disease (PD) is one of the most common neurodegenerative disorders, affecting more than 1% of the global population over 65 years of age, and its incidence is expected to double by 2030 [1]. The etiology of PD involves a complex interplay between genetic and environmental risk factors. Several causative genes have been identified that shed light on the mechanisms underlying PD, including mitochondrial dysfunction, dysregulation of protein degradation, and abnormal intracellular cargo or organelle trafficking [2]. Current treatments are mainly symptomatic, leading to an unmet medical need for mechanism-based therapy for PD.

In neurons, the cytoskeleton is instrumental in establishing and maintaining architecture and function. Microtubules, in association with dyneins and kinesins, regulate intracellular organelle transport that are critical for neurite morphogenesis [3]. Alterations of microtubule assembly or post-translational tubulin modification may contribute to PD pathogenesis [4]. The dominant mutation in leucine-rich repeat kinase (LRRK2), p.Gly2019Ser (G2019S), is the most common mutation causing familial and sporadic forms of PD. This mutation is associated with cytoskeletal component dysfunction, influencing vesicular biogenesis, organelle trafficking, and synaptic signaling [5–7]. LRRK2 carrying the G2019S mutation activates glycogen synthase kinase 3β (GSK3β), enhancing tau protein phosphorylation and resulting in cytoskeleton instability and neurite degeneration [8]. Furthermore, Lrrk2 can directly bind to three β-tubulin isoforms at the luminal face of microtubules, suppressing α-tubulin acetylation, decreasing kinesin binding and impairing the microtubule-based endosome, Golgi, and mitochondrial trafficking [5, 9, 10]. These findings suggest that microtubule disruption leads to defective axonal transport, resulting in PD pathology. Thus, a strategy to stabilize microtubule integrity or promote cytoskeleton-based cargo trafficking may be beneficial for *LRRK2* parkinsonism and promising for PD treatment.

Statins are competitive inhibitors of 3-hydroxy-3-methylglutaryl coenzyme A (HMG-CoA) reductase (HMGR) and were recently found to have neuroprotective effects [11–13]. Statins have been shown to reduce intraneuronal α-synuclein aggregations in animal PD models and restore neurite degeneration by augmenting the Akt/NRF2 pathway and inhibiting downstream GSK3β activity in transgenic *LRRK2-G2019S* Drosophila and knock-in mouse models [14]. Histone deacetylase (HDAC) inhibitors can relax chromatin through histone acetylation, promoting the expression of multiple genes conducive to neuroprotection. HDAC inhibitors have been shown to combat aging-associated cognitive decline, as well as dopaminergic neuron loss and PD risk [15, 16], suggesting that they may protect against neurodegenerative disorders.

Given the multi-faceted mechanism underlying PD pathogenesis, it is likely that combination or multi-target therapies will be promising in halting PD progression. As both HMGR and HDAC inhibitors exhibit neuroprotective potential, we have designed and synthesized a dual-acting compound, statin hydroxamate (JMF3086), that simultaneously inhibits HMGR and class I and II HDACs [17]. In the present study, we examined the effects of JMF3086 on PD-related neurodegeneration in primary hippocampal and dopaminergic neurons from transgenic *LRRK2-G2019S* mice and Drosophila models [18]. We also employed stably transfected *LRRK2-G2019S* SH-SY5Y human dopaminergic cells to elucidate the mechanisms of action of JMF3086. Our results demonstrate that this dual inhibitor exerted beneficial effects on the neurodegeneration in *LRRK2* parkinsonism.

## RESULTS

### JMF3086 protected against age-dependent dopaminergic neuron loss in a transgenic *LRRK2-G2019S* Drosophila model

We previously developed a transgenic *LRRK2-G2019S* Drosophila model that recapitulates several PD phenotypes, including age-dependent dopaminergic neuron degeneration and locomotor disability [18]. Dopaminergic neuron clusters that express tyrosine hydroxylase (TH) are present in each Drosophila adult brain hemisphere (Supplementary Figure 1A, 1B) [19]. Overexpression of the Lrrk2 protein carrying the G2019S mutation led to dopaminergic neuron degeneration in aged flies (Supplementary Figure 1C; statistics in Supplementary Figure 1G) [18]. We administered JMF3086 at 10 or 20 mg/ml during the first larval stage, and then immunostained the brains of adult flies (4 weeks of age) emerging from pupae with anti-TH antibodies. Lovastatin, SAHA, and JMF3086 at 20 mg/ml all exerted beneficial effects, protecting against dopaminergic neuron loss in aged *LRRK2-G2019S* flies compared to DMSO solvent control (Supplementary Figure 1D–1F, statistics in Supplementary Figure 1G).

### JMF3086 restored neurite degeneration in primary hippocampal and dopaminergic neurons from transgenic *LRRK2-G2019S* mice

**Figure 1:**
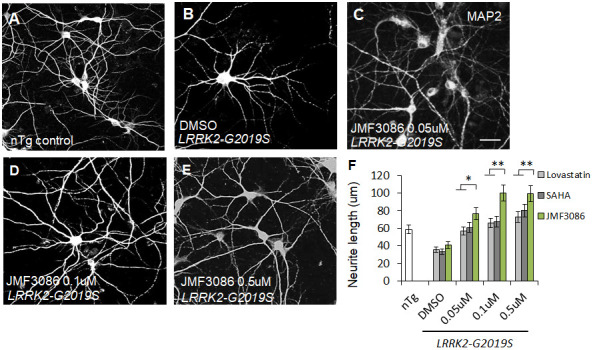
**JMF3086 synergistically mitigates neurite degeneration in primary hippocampal neurons from transgenic *LRRK2-G2019S* mice compared to treatment with lovastatin or SAHA.** (**A**–**E**) Representative images showing cultured primary hippocampal neurons (DIV 14) from (**A**) non-transgenic littermate control (nTg) and (**B**–**E**) *LRRK2-G2019S* pups treated with (**B**) vehicle DMSO or (**C**–**E**) JMF3086 at different concentrations. Neurites were stained with anti-MAP2 antibody. Scale bar, 100 μm. (**F**) Quantitative analysis of mean total neurite length for the neurons described in A–E, with treatment using different concentrations of lovastatin and SAHA. We analyzed 50–100 neurons for each genotype or treatment condition. Data represent mean ± SEM. *P<0.05, **P<0.01.

*LRRK2-G2019S* expression is associated with shortened neurites in both primary hippocampal neuron cultures and dentate gyrus granule cells from mice [8, 20–22]. As neurite shortening is ubiquitous in *LRRK2-G2019S* Drosophila and mammalian models, neurite morphology was considered a surrogate phenotype for examining the neuroprotective effects of JMF3086. Primary hippocampal neurons were harvested from transgenic *LRRK2-G2019S* mice and cultured for at least 14 days *in vitro* (DIV14). To distinguish between glia and neurons, we stained neurons for microtubule-associated protein 2 (MAP2). Consistent with previous studies [8], the length and complexity of neurite branches were significantly reduced in *LRRK2-G2019S* mice compared to transgenic wild-type (*LRRK2*-WT) mice and non-transgenic littermate controls (Supplementary Figures 2A–2C, statistics in Supplementary Figure 2D). The mean neurite length of the *LRRK2-G2019S* hippocampal neurons was 36.9±4.6 μm. JMF3086 exerted beneficial effects with neurite lengths of 76.8±8.0 μm, 100.3±9.9 μm, and 99.3±9.5 μm at 0.05, 0.1, and 0.5 μM, respectively (Figure 1A–1E, statistics in Figure 1F). These beneficial effects on neurite length were more prominent than those obtained with the same concentrations of lovastatin (56.5±5.9 μm, 67.1±6.0 μm, and 72.8±8.1 μm, respectively) or SAHA treatment (60.9±7.1 μm, 67.1±5.9 μm, and 79.9±8.2 μm, respectively; Figure 1F).

**Figure 2:**
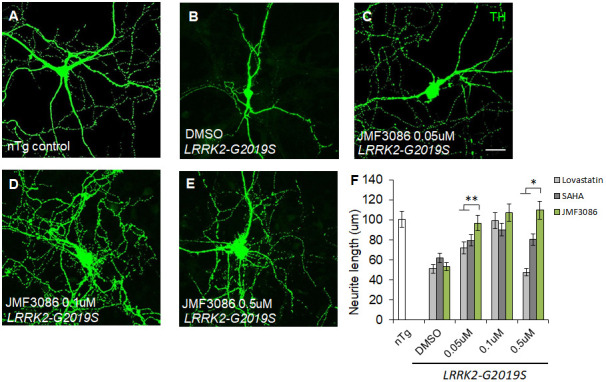
**JMF3086 synergistically promotes neurite arborization in primary nigral TH-positive neurons from transgenic *LRRK2-G2019S* mice compared to treatment with lovastatin or SAHA.** (**A**–**E**) Representative images showing cultured primary nigral TH-positive neurons (DIV 14) from (A) non-transgenic littermate control (nTg) and (**B**–**E**) *LRRK2-G2019S* pups treated with (**B**) vehicle DMSO or (**C**–**E**) different concentrations of JMF3086. Neurites were stained with anti-TH antibody. Scale bar, 100 μm. (**F**) Quantitative analysis of mean total neurite length for the TH(+) neurons described in A–E, with treatment using different concentrations of lovastatin and SAHA. We analyzed 30–50 neurons for each genotype or treatment condition. Data represent mean ± SEM. *P<0.05, **P<0.01.

As PD is a primary dopaminergic neurodegenerative disorder, we further examined the effects of JMF3086 on nigral TH-positive dopaminergic neurons isolated from transgenic *LRRK2-G2019S* mice. Consistent with the findings in primary hippocampal neurons, we observed neurite shortening in TH-positive nigral neurons from mice expressing Lrrk2 carrying the G2019S mutation (mean neurite length 53.3±6.0 μm) compared to transgenic *LRRK2-WT* mice and littermate controls (Supplementary Figure 2E–2G, statistics in Supplementary Figure 2H). JMF3086 had beneficial effects to restore neurite length to 96.8±10.1 μm, 107.3±9.3 μm, and 109.7±12.4 μm at 0.05, 0.1, and 0.5 μM, respectively (Figure 2A–2E, statistics in Figure 2F). Comparatively, we observed modest neuroprotective effects in terms of the neurite length of TH(+) nigral neurons after treatment with lovastatin (72.1±8.1 μm, 99.2±8.8 μm, and 47.6±5.2 μm, respectively) or SAHA (79.6±8.0 μm, 90.1±9.9 μm, and 80.6±8.1 μm, respectively; Figure 2F). These data reinforced that JMF3086 had significantly beneficial effects on halting neurite degeneration in *LRRK2-G2019S* parkinsonism.

### JMF3086 inhibited the ERK1/2 phosphorylation pathway and decreased apoptosis

**Figure 3:**
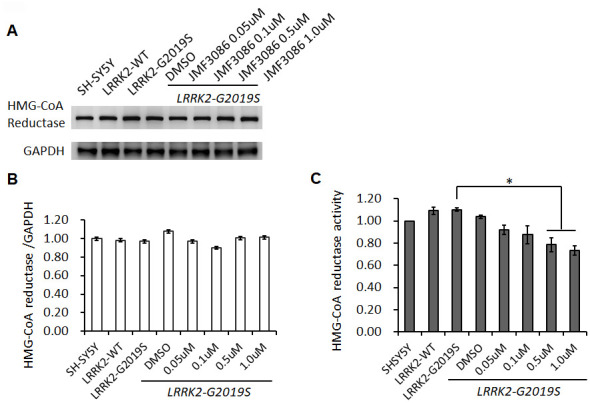
**Effects of different concentrations of JMF3086 on HMG-CoA reductase expression and enzymatic activity in SH-SY5Y cells stably transfected with *LRRK2-G2019S.*** (**A**) Western blot of HMG-CoA reductase protein expression after treatment with different concentrations of JMF3086. (**B**) Quantification of drug effects on the ratio of HMG-CoA reductase to GAPDH. (**C**) Quantification of HMG-CoA reductase enzymatic activity after treatment with different concentrations of JMF3086. Experiments were repeated in triplicate, and the ratio was compared to SH-SY5Y control cells without transgenes and drug treatment. The relative HMG-CoA reductase enzymatic activity of *LRRK2-WT*, *LRRK2-G2019S* without treatment, or after treatment with DMSO solvent, 0.05 μM, 0.1 μM, 0.5 μM, and 1.0 μM JMF3086 was 1.09±0.03, 1.10±0.02, 1.04±0.02, 0.92±0.04, 0.88±0.08, 0.78±0.06, and 0.74±0.03, respectively. *P*=0.04 for *LRRK2-G2019S* vs. *LRRK2-G2019S* with 0.5 μM or 1.0 μM JMF3086, one-way ANOVA. All neurons were treated for 48 hours and then lysed. Equal amounts of protein lysate were subjected to SDS-PAGE and the proteins analyzed by Western blotting. Immunoblots were probed with the indicated antibodies. Data represent mean ± SEM. *P<0.05, **P<0.01.

To examine the action mechanism of JMF3086 in mitigating neurite degeneration, we used the SH-SY5Y human dopaminergic neuronal cell line stably expressing *LRRK2-G2019S* [20]. JMF3086 did not affect HMGR expression at 0.05–1.0 μM (Figure 3A, 3B), but dose-dependently inhibited its enzyme activity (Figure 3C). Such treatment also inhibited HDAC activity, mainly for class I and II HDACs, with an *in vitro* IC50 in the nanomolar range as our previous report [17].

**Figure 4:**
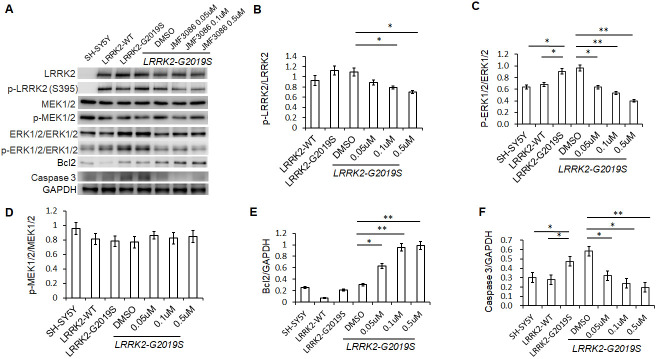
**JMF3086 inhibited Lrrk2 MAPKKK activity and down-regulated ERK1/2 signaling pathways in SH-SY5Y cells stably expressing *LRRK2-G2019S*.** (**A**) Western blot of total and phosphorylated target proteins, including Lrrk2, MEK1/2, ERK1/2, pro-apoptotic caspase 3, and anti-apoptotic Bcl2 after treatment with different concentrations of JMF3086. (**B**) Quantification of the effects of JMF3086 on the ratio of p-Lrrk2 to total Lrrk2. The relative expression of p-Lrrk2 to total Lrrk2 in *LRRK2-WT* and *LRRK2-G2019S* after treatment with DMSO solvent, 0.05 μM, 0.1 μM, and 0.5 μM JMF3086 was 0.92±0.08, 1.13±0.09, 1.09±0.08, 0.88±0.05, 0.78±0.03, and 0.70±0.04, respectively. *P*=0.09 for *LRRK2-WT* vs. *LRRK2-G2019S*; *P*=0.07 for *LRRK2-G2019S* with DMSO solvent vs. *LRRK2-G2019S* with 0.05 μM JMF3086; *P*=0.04 for *LRRK2-G2019S* with DMSO solvent vs. *LRRK2-G2019S* with 0.1 μM JMF3086; *P*=0.02 for *LRRK2-G2019S* with DMSO solvent vs. *LRRK2-G2019S* with 0.5 μM JMF3086, all one-way ANOVA. (**C**) Quantification of the effects of JMF3086 on the ratio of p-ERK1/2 to total ERK1/2. The relative expression of p-ERK1/2 to total ERK1/2 in SH-SY5Y controls, *LRRK2-WT,* and *LRRK2-G2019S* after treatment with DMSO solvent, 0.05 μM, 0.1 μM, and 0.5 μM JMF3086 was 0.63±0.05, 0.67±0.06, 0.92±0.10, 0.96±0.11, 0.63±0.09, 0.53±0.07, and 0.39±0.05, respectively. *P*=0.04 for SH-SY5Y controls or *LRRK2-WT* vs. *LRRK2-G2019S*; *P*=0.02 for *LRRK2-G2019S* with DMSO solvent vs. *LRRK2-G2019S* with 0.05 μM JMF3086; *P*=0.009 for *LRRK2-G2019S* with DMSO solvent vs. *LRRK2-G2019S* with 0.1 μM JMF3086; *P*=0.007 for *LRRK2-G2019S* with DMSO solvent vs. *LRRK2-G2019S* with 0.5 μM JMF3086, all one-way ANOVA. (**D**) Quantification of the effects of JMF3086 on the ratio of p-MEK1/2 to total MEK1/2. The relative expression of p-MEK1/2 to total MEK1/2 in SH-SY5Y controls, *LRRK2-WT,* and *LRRK2-G2019S* after treatment with DMSO solvent, 0.05 μM, 0.1 μM, and 0.5 μM JMF3086 was 0.95±0.08, 0.81±0.07, 0.78±0.08, 0.77±0.08, 0.86±0.05, 0.82±0.08, and 0.85±0.08, respectively. (**E**) Quantification of the effects of JMF3086 on the ratio of Bcl2 to GAPDH. The relative expression of Bcl2 to GAPDH in SH-SY5Y controls, *LRRK2-WT,* and *LRRK2-G2019S* after treatment with DMSO solvent, 0.05 μM, 0.1 μM, and 0.5 μM JMF3086 was 0.25±0.02, 0.07±0.01, 0.21±0.02, 0.30±0.01, 0.62±0.10, 0.95±0.12, and 0.98±0.14, respectively. *P*=0.02 for *LRRK2-G2019S* with DMSO solvent vs. *LRRK2-G2019S* with 0.05 μM JMF3086; *P*=0.009 for *LRRK2-G2019S* with DMSO solvent vs. *LRRK2-G2019S* with 0.1 μM JMF3086; *P*=0.008 for *LRRK2-G2019S* with DMSO solvent vs. *LRRK2-G2019S* with 0.5 μM JMF3086, all one-way ANOVA. (**F**) Quantification of the effects of JMF3086 on the ratio of caspase-3 to GAPDH. The relative expression of caspase-3 to GAPDH in SH-SY5Y controls, *LRRK2-WT,* and *LRRK2-G2019S* after treatment with DMSO solvent, 0.05 μM, 0.1 μM, and 0.5 μM JMF3086 was 0.30±0.07, 0.28±0.06, 0.47±0.08, 0.58±0.05, 0.32±0.07, 0.24±0.08, and 0.20±0.08, respectively. *P*=0.04 for SH-SY5Y controls or *LRRK2-WT* vs. *LRRK2-G2019S*; *P*=0.03 for *LRRK2-G2019S* with DMSO solvent vs. *LRRK2-G2019S* with 0.05 μM JMF3086; *P*=0.02 for *LRRK2-G2019S* with DMSO solvent vs. *LRRK2-G2019S* with 0.1 μM JMF3086; *P*=0.009 for *LRRK2-G2019S* with DMSO solvent vs. *LRRK2-G2019S* with 0.5 μM JMF3086, all one-way ANOVA. All neurons were treated for 48 h and then lysed. Equal amounts of protein lysate were subjected to SDS-PAGE and the proteins analyzed by Western blotting. Immunoblots were probed with the indicated antibodies. All experiments were repeated three times. Data represent mean ± SEM. *P<0.05, **P<0.01.

Lrrk2 is a member of the ROCO protein family and this multi-domain protein contains both a GTPase (ROC domain) and a mitogen-activated protein kinase kinase kinase (MAPKKK) domain. The G2019S mutation is located in its catalytic MAPKKK domain and aberrantly increases the kinase activity. One downstream mediator of *LRRK2-G2019S* neurodegeneration is the up-regulation of mitogen-activated protein kinases 3 and 1 (also termed ERK1/2) [21]. As ERK1/2 regulates neuronal plasticity and cell survival, increased ERK1/2 phosphorylation has been shown to be a common pathway contributing to neuronal susceptibility in PD [22]. Lrrk 2 is a homodimer with auto-phosphorylation activity, phosphorylation at residue S395 is used to indicate Lrrk2 kinase activity [23]. We found treatment with JMF3086 significantly decreased the ratio of p-Lrrk2 to total Lrrk2 in a dose-dependent manner (Figure 4A, statistics in Figure 4B). We also observed an increased ratio of p-ERK1/2 to total ERK1/2 in SH-SY5Y neurons expressing *LRRK2-G2019S*. Notably, JMF3086 dose-dependently decreased the ratio of p-ERK1/2 to total ERK1/2, which was accompanied by decreased apoptotic caspase-3 and increased anti-apoptotic Bcl-2 (Figure 4A, statistics in Figure 4C, 4E, 4F), correlating with the rescue of neurite shortening in primary hippocampal and nigral TH-positive neurons (Figures 1F and 2F). However, the ratio of phosphorylated MAPKKK (p-MEK1/2) to MEK1/2, which is an up-stream kinase of ERK1/2, did not change in LRRK2-G2019S cells treated with different concentrations of JMF3086 (Figure 4A, statistics in Figure 4D).

### JMF3086 decreased GSK3β activity and tau phosphorylation by activating the Akt pathway

**Figure 5:**
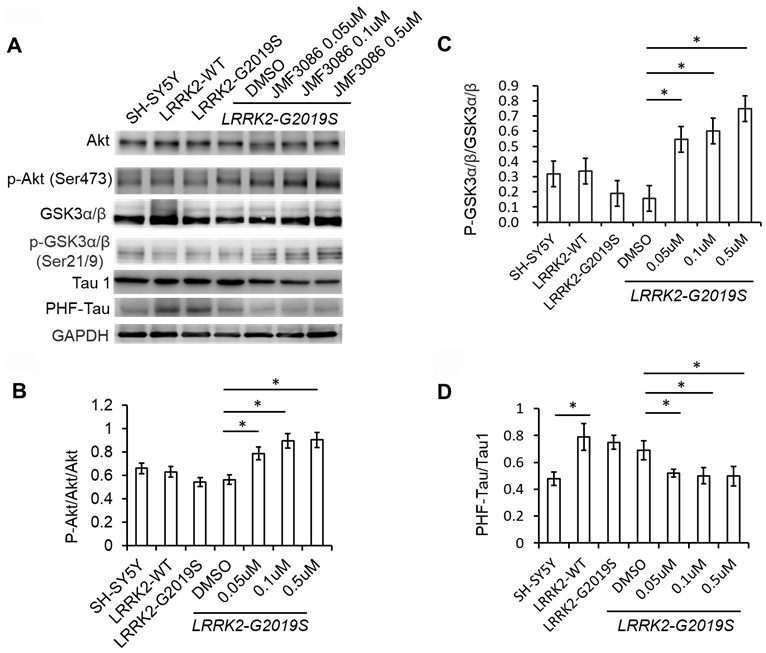
**JMF3086 inhibited GSK3b-tau phosphorylation by activating Akt phosphorylation and decreasing neuronal apoptosis cascade in *LRRK2-G2019S* neurons.** (**A**) Western blot of total and phosphorylated Akt, GSK3b, and tau after treatment with different concentrations of JMF3086. (**B**–**D**) Quantification of the effects of JMF3086 on the ratios of (**B**) p-Akt to total Akt, (**C**) p-GSK3α/β (Ser21/9) to total GSK3a/b, and (**D**) PHF-Tau to Tau1 in stably transfected *LRRK2-G2019S* SH-SY5Y cells. (**B**) The relative expression of p-Akt to total Akt in SH-SY5Y controls, *LRRK2-WT,* and *LRRK2-G2019S* after treatment with DMSO solvent, 0.05 μM, 0.1 μM, and 0.5 μM JMF3086 was 0.66±0.08, 0.63±0.07, 0.55±0.05, 0.56±0.06, 0.78±0.10, 0.89±0.09, and 0.91±0.10, respectively. *P*=0.04 for *LRRK2-G2019S* with DMSO solvent vs. *LRRK2-G2019S* with 0.05 μM JMF3086; *P*=0.03 for *LRRK2-G2019S* with DMSO solvent vs. *LRRK2-G2019S* with 0.1 μM JMF3086; *P*=0.03 for *LRRK2-G2019S* with DMSO solvent vs. *LRRK2-G2019S* with 0.5 μM JMF3086, all one-way ANOVA. (**C**) The relative expression of p-GSK3α/β (Ser21/9) to total GSK3a/b in SH-SY5Y controls, *LRRK2-WT,* and *LRRK2-G2019S* after treatment with DMSO solvent, 0.05 μM, 0.1 μM, and 0.5 μM JMF3086 was 0.32±0.07, 0.34±0.08, 0.20±0.08, 0.16±0.09, 0.55±0.10, 0.60±0.11, and 0.75±0.10, respectively. *P*=0.03 for *LRRK2-G2019S* with DMSO solvent vs. *LRRK2-G2019S* with 0.05 μM JMF3086; *P*=0.02 for *LRRK2-G2019S* with DMSO solvent vs. *LRRK2-G2019S* with 0.1 μM JMF3086; *P*=0.02 for *LRRK2-G2019S* with DMSO solvent vs. *LRRK2-G2019S* with 0.5 μM JMF3086, all one-way ANOVA. (**D**) The relative expression of PHF-Tau to Tau1 in SH-SY5Y controls, *LRRK2-WT,* and *LRRK2-G2019S* after treatment with DMSO solvent, 0.05 μM, 0.1 μM, and 0.5 μM of JMF3086 was 0.48±0.05, 0.79±0.10, 0.75±0.05, 0.65±0.08, 0.52±0.03, 0.50±0.06, and 0.49±0.07, respectively. *P*=0.02 for SH-SY5Y controls or *LRRK2-WT* vs. *LRRK2-G2019S*; *P*=0.04 for *LRRK2-G2019S* with DMSO solvent vs. *LRRK2-G2019S* with 0.05 μM JMF3086; *P*=0.03 for *LRRK2-G2019S* with DMSO solvent vs. *LRRK2-G2019S* with 0.1 μM JMF3086; *P*=0.03 for *LRRK2-G2019S* with DMSO solvent vs. *LRRK2-G2019S* with 0.5 μM JMF3086, all one-way ANOVA. All neurons were treated for 48 h and then lysed. Equal amounts of protein lysate were subjected to SDS-PAGE and the proteins e analyzed by Western blotting. Immunoblots were probed with the indicated antibodies. All experiments were repeated three times. Data represent mean ± SEM. *P<0.05, **P<0.01.

In addition to the ERK1/2 pathway, the phosphoinositide-3-kinase (PI3K)/Akt signaling pathway also regulates neuronal survival, neurite outgrowth, and arborization [24]. The ERK1/2 and PI3K/Akt pathways engage in positive or negative cross-talk to regulate neuron survival [24]. The glycogen synthase kinase 3β (GSK3β) signaling cascade is downstream of the PI3K/Akt pathway and has been implicated in the pathogenesis of *LRRK2-G2019S* neurodegeneration through tau protein hyper-phosphorylation [18, 25]. GSK3β is constitutively active in resting cells and inhibited by Ser9 phosphorylation via Akt. We examined the effect of JMF3086 on Akt phosphorylation (p-AktSer473) in *LRRK2-G2019S* SH-SY5Y neurons. JMF3086 dose-dependently induced Akt phosphorylation, accompanied by an increase in p-GSK3βSer9 without changes in the total GSK3β levels (Figure 5A, statistics in Figure 5B, 5C). This indicates that JMF3086 inhibited GSK3β activity. Consistently, the phosphorylated paired helical tau (PHF-tau) level was also decreased without changes in its total expression (Figure 5A, statistics in Figure 5D).

Overall, these findings suggest that, in addition to down regulating the ERK1/2 pathway, JMF3086 also activated Akt, reduced GSK3β activity and tau phosphorylation.

### JMF3086 increased α-tubulin acetylation and kinesin expression

Both microtubule integrity and cytoskeleton-based cargo trafficking are crucial for maintaining neuronal architecture and function. Altered post-translational modifications, such as tau hyper-phosphorylation or α**-**tubulin deacetylation, may disrupt microtubule assembly and cytoskeleton-based cargo trafficking, contributing to PD pathogenesis [26].

JMF3086 inhibits the activity of class I/II HDACs, including HDAC6 [17], which is exclusively localized in the cytoplasm, where it associates with microtubules and co-localizes with microtubule motor proteins [27]. Inhibition of HDAC6 activity increases α-tubulin acetylation and enhances its binding to kinesin to stabilize microtubules [28, 29]. JMF3086 increased α-tubulin acetylation in *LRRK2-G2019S* SH-SY5Y cells. Tubulin acetylation has been reported to promote microtubule interaction with kinesin-1 (Kif 5), inducing direct kinesin-1-based cargo transport to growing neurites in developing neurons and axons in mature neurons [30]. Consistently, JMF3086 enhanced kinesin-1 expression in *LRRK2-G2019S* neurons that reinforced its promotion on kinesin-1 binding with microtubules to transport cargo to axons.

**Figure 6:**
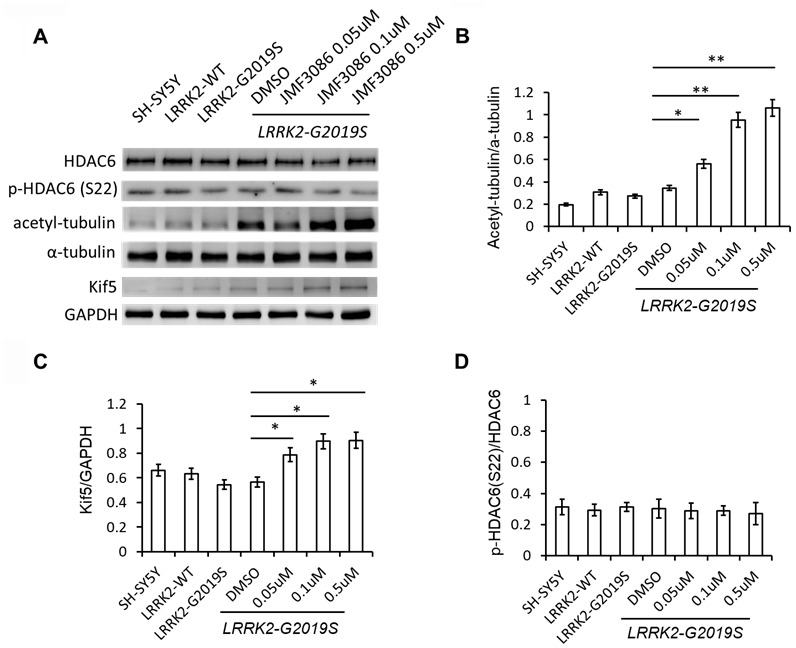
**JMF3086 increased tubulin acetylation and kinesin-1 (kif5) expression.** (**A**) Western blot of total and phosphorylated HDAC6, a-tubulin, acetyl-tubulin, and kinesin kif5 protein in stably transfected *LRRK2-G2019S* SH-SY5Y cells after treatment with different concentrations of JMF3086. (**B**) Quantification of the effects of JMF3086 on the ratio of acetyl-tubulin to total a-tubulin. The relative expression of acetyl-tubulin to total a-tubulin in SH-SY5Y controls, *LRRK2-WT,* and *LRRK2-G2019S* after treatment with DMSO solvent, 0.05 μM, 0.1 μM, and 0.5 μM JMF3086 was 0.19±0.02, 0.31±0.05, 0.27±0.03, 0.35±0.04, 0.56±0.08, 0.95±0.10, and 1.01±0.13, respectively. *P*=0.04 for *LRRK2-G2019S* with DMSO solvent vs. *LRRK2-G2019S* with 0.05 μM JMF3086; *P*=0.009 for *LRRK2-G2019S* with DMSO solvent vs. *LRRK2-G2019S* with 0.1 μM JMF3086; *P*=0.008 for *LRRK2-G2019S* with DMSO solvent vs. *LRRK2-G2019S* with 0.5 μM JMF3086, all one-way ANOVA. (**C**) Quantification of the effects of JMF3086 on Kif5 expression. The expression of Kif5 relative to GAPDH in SH-SY5Y controls, *LRRK2-WT,* and *LRRK2-G2019S* after treatment with DMSO solvent, 0.05 μM, 0.1 μM, and 0.5 μM JMF3086 was 0.66±0.05, 0.63±0.06, 0.54±0.04, 0.56±0.05, 0.79±0.08, 0.89±0.09, and 0.91±0.10, respectively. *P*=0.04 for *LRRK2-G2019S* with DMSO solvent vs. *LRRK2-G2019S* with 0.05 μM JMF3086; *P*=0.03 for *LRRK2-G2019S* with DMSO solvent vs. *LRRK2-G2019S* with 0.1 μM JMF3086; *P*=0.03 for *LRRK2-G2019S* with DMSO solvent vs. *LRRK2-G2019S* with 0.5 μM JMF3086, all one-way ANOVA. (**D**) Quantification of the effects of JMF3086 on the ratio of p-HDAC6 (Ser22) to total HDAC6. The relative expression of p-HDAC6 to total HDAC6 in SH-SY5Y controls, *LRRK2-WT,* and *LRRK2-G2019S* after treatment with DMSO solvent, 0.05 μM, 0.1 μM, and 0.5 μM JMF3086 was 0.31±0.05, 0.29±0.04, 0.31±0.03, 0.30±0.06, 0.29±0.05, 0.28±0.03, and 0.27±0.07, respectively. All experiments were repeated three times. Data represent mean ± SEM. *P<0.05, **P<0.01.

Recently, HDAC6 was identified as a novel Lrrk2 substrate [31]. Lrrk2 has been shown to phosphorylate HDAC6 on Ser22, enhancing its interaction with dynein to promote the recruitment of ubiquitinated proteins forming aggresomes [31]. However, we did not detect significant changes of phospho-HDAC6 on Ser22 in SH-SY5Y cells expressing *LRRK2-G2019S*, and JMF3086 did not alter the ratio of p-HDAC6 to total HDAC6, either (Figure 6A, statistics in Figure 6D).

### JMF3086 promoted mitochondrial transport in axons to mitigate neurite degeneration in *LRRK2-G2019S* neurons

**Figure 7:**
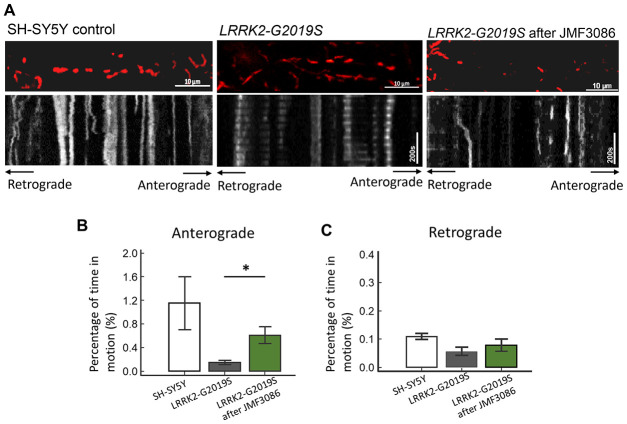
**Enhanced expression of acetyl-tubulin by JMF3086 promoted mitochondrial antegrade movement in the axons of SH-SY5Y cells carrying *LRRK2-G2019S*.** Mitochondrial movement was labeled by mito-GFP in representative axons. (**A**) Kymographs depicting mitochondrial motility. A segment of axon was imaged continuously for 3 hours with a short break to allow for drug administration. Images were acquired at 10-second intervals. Left, SH-SY5Y control cells without treatment. Middle, *LRRK2-G2019S* cells without treatment. Right, *LRRK2-G2019S* cells treated with 0.5 μM JMF3086 for 24 hours. (**B**, **C**) From kymographs in (**A**), we determined and averaged the percentage of time that each mitochondrion was in motion in an antegrade direction to the terminal neurite (**B**) or retrograde to the soma (**C**). *n* = 30–50 mitochondria from nine axons per genotype and treatment. Data represent mean ± SD. *P<0.05, **P<0.01.

Disrupted axonal transport is an early feature of neurodegenerative disorders, including PD [32]. *PINK1* and *Parkin* mutations affecting axonal mitochondrial transport have been shown to cause the majority of cases of early-onset PD [33, 34]. Therefore, whether JMF3086 promotes axonal mitochondrial transport in *LRRK2-G2019S* neurons was examined using time-lapse fluorescence microscopy to trace the movement of RFP-tagged mitochondria (mito-RFP). Compared to control SH-SY5Y neurons, the *LRRK2-G2019S* mutant exhibited reduced dynamics of mitochondrial movement in both the anterograde and retrograde directions (Figure 7A, statistics in Figure 7B, 7C). Notably, 0.5 μM JMF3086 significantly restored antegrade mitochondrial movements to axons (Kymographs in Figure 7A and statistics in Figure 7B). The percentage of moving time in *LRRK2-G2019S* cells improved from 0.15±0.07% to 0.62±0.27% after JMF3086 treatment (*P*=0.04), those in control SH-SY5Y cells was 1.15±0.90%. These findings correspond with the neurite length analysis in *LRRK2-G2019S* SH-SY5Y cells treated with JMF3086 (Supplementary Figure 3). Confocal microscopy revealed shortened neurites in *LRRK2-G2019S*-expressing cells (50.3±10.2 μm) compared to wild-type neurons (80.3±11.2 μm; *P*=0.009), and JMF3086 restored the neurite retraction phenotype in *LRRK2-G2019S* cells (0.05 μM: 53.5±13.2 μm; 0.1 μM: 62.5±10.9 μm; and 0.5 μM: 71.3±12.8 μm; Supplementary Figure 3A–3E, statistics in Supplementary Figure 3F). These results demonstrate that JMF3086 elicited microtubule acetylation to restore axonal mitochondrial transport defects in the *LRRK2-G2019S* mutant as well as its neurite degeneration.

## DISCUSSION

**Figure 8:**
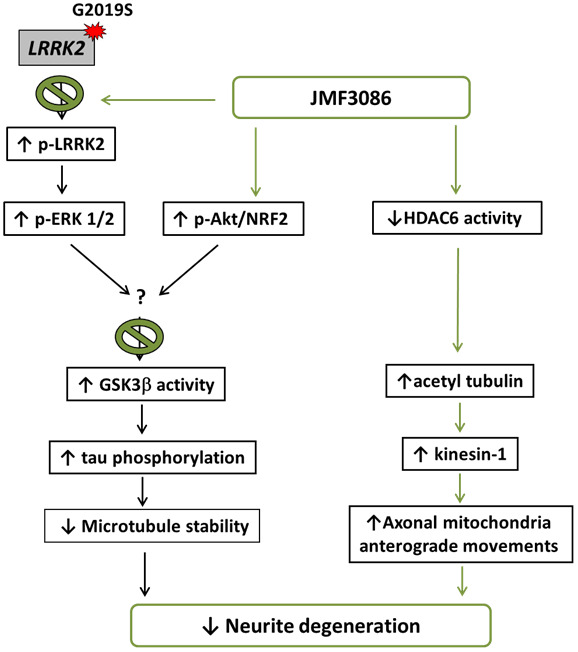
**Schematic representation of a potential mechanism for the rescue of *LRRK2-G2019S* neurite degeneration by JMF3086.**

In the present study, we demonstrated that JMF3086, a dual inhibitor targeting HMGR and HDAC, significantly improved neurite degeneration in *LRRK2-G2019S* parkinsonism models both *in vivo* and *in vitro*. JMF3086 protected age-dependent dopaminergic neuron loss in transgenic *LRRK2-G2019S* flies and rescued neurite degeneration in primary hippocampal and nigral TH-positive dopaminergic neurons isolated from *LRRK2-G2019S* mice. Human dopaminergic neuronal cell lines stably expressing *LRRK2-G2019S* revealed multi-targeted mechanisms, maintaining microtubule stability via down-regulation of ERK1/2 phosphorylation, activation of the Akt pathway, and downregulation of GSK3β activity to decrease tau phosphorylation, and promoting microtubule acetylation and kinesin-1 expression to facilitate mitochondrial antegrade transport to axons. Figure 8 presents a schematic overview of these mechanisms.

The motor manifestations of PD are largely caused by the degeneration of dopaminergic neurons in the substantia nigra pars compacta projecting to the striatum, where dopaminergic neuron axons extensively branch, generating a dense lattice that provides dopaminergic innervation to the medium spiny neurons. Each human nigral dopaminergic neuron gives rise to 1–2.5 million synapses in the striatum, with the total axon length exceeding 4 m [35]. As the demand for cellular trafficking machinery in nigral dopaminergic neurons is far greater than that in other neurons. Impaired cellular trafficking would therefore tremendously affect nigral dopaminergic neurons. Among microtubule-based cargo trafficking, axonal mitochondrial trafficking is vital for proper neuronal function, and its perturbation is associated with PD [36]. Kinesin-1 is a motor protein that carries cargo, including mitochondria, toward the plus end of microtubules. Tubulin acetylation reportedly promotes binding with kinesin-1 to facilitate the trafficking of kinesin-1-dependent cargo, including mitochondria [37]. HDAC6 inhibition increases α-tubulin acetylation. Here, we found that JMF3086 dose-dependently increases the acetyl-tubulin level in parallel with enhanced kinesin-1 expression, promoting axonal anterograde movement of mitochondria and decreasing neurite degeneration in *LRRK2-G2019S* neurons. Consistent with our findings, one recent study using transgenic *Drosophila* as a PD model demonstrated that Lrrk2 containing pathogenic mutations inhibits axonal transport, causing locomotor deficits *in vivo* [38]. Treatment with deacetylase inhibitors increased microtubule acetylation to restore axonal transport, and knockdown of HDAC6 rescued both axonal transport and locomotor behavior [38]. Thus, our findings reinforce the link between impaired microtubule acetylation in LRRK2 parkinsonism and increased α-tubulin acetylation through inhibiting HDAC6 activity revealed a beneficial effect in mitigating neuronal degeneration in PD.

ERK1/2 belongs to the mitogen-activated protein kinase (MAPK) family and regulates diverse cellular functions, including growth, division, differentiation, and death. ERK1/2 activation has been observed in midbrain neurons from human PD patients. Delayed ERK1/2 activation attributed to mitochondrial dysfunction has been reported in acute 6-hydroxydopamine (6-OHDA) and chronic 1-methyl-4-phenyl-1,2,3,6-tetrahydropyridine (MPTP) mouse models of PD [39]. These observations suggest that ERK1/2 activation is involved in PD pathobiology. We and other groups have shown that ERK1/2 dysregulation is a downstream mediator in mutant *LRRK2-G2019S* *Drosophila* and cellular models [13, 40]. The *LRRK2-G2019S* mutation leads to increased phospho-ERK1/2 levels and promotes neurite retraction [21]. ERK1/2 inhibition ameliorated apoptosis of iPSCs derived from PD patients harboring *LRRK2-G2019S* mutation [41]. In the present study, we observed that the increased phospho-ERK1/2 in *LRRK2-G2019S* neurons is dose-dependently inhibited by JMF3086. Accordingly, JMF3086 rescued neurite degeneration. These data suggest that inhibition of the ERK1/2-activated pathway in *LRRK2-G2019S* neurons contributes to the rescue of neurite degeneration by JMF3086.

GSK3β activity is also reported to be associated with *LRRK2-G2019S* parkinsonism and sporadic PD [18, 42]. In contrast to other kinases, GSK3β is constitutively active in resting cells and inhibited by phosphorylation of its Ser9 residue by upstream kinases, such as Akt. GSK3β is an upstream kinase that phosphorylates tau, which is involved in the establishment and maintenance of microtubule stability and neuronal morphology [43]. We and others have found that *LRRK2-G2019S* induces dendrite degeneration by recruiting auto-activated GSK3β to increase tau phosphorylation and microtubule fragmentation in dendrites [18, 25]. In addition, lovastatin can inhibit GSK3β activity by activating Akt [13]. Lovastatin-mediated neuroprotection is dependent on Akt signaling and inhibition of GSK3β activity [13]. In current study, we demonstrated that JMF3086 activated Akt and inhibited the GSK3β pathway, exerting neuroprotective activity by preventing microtubule instability and, thus, mitigating the neurite degeneration in *LRRK2-G2019S* neurons.

Although monotherapy is effective for numerous diseases, it may be insufficient for producing significant improvements in complex neurodegenerative disorders, such as PD. Combination therapy or multi-target drugs may be more beneficial in mitigating PD progression. Multicomponent drug cocktails can have several drawbacks, including complex pharmacokinetics, unpredictable drug–drug interactions, and formulation problems. Here, we demonstrated that the dual-acting compound JMF3086 derived from lovastatin and SAHA beneficially rescued neurite degeneration in a *LRRK2-G2019S* parkinsonism model. JMF3086 acts through multiple pathways to enhance microtubule stability, increase tubulin acetylation, and enhance kinesin-1 expression to promote axonal mitochondrial transport. The beneficial effect of JMF3086 suggests a potential clinical implication for future treatments of PD.

## MATERIALS AND METHODS

### *Drosophila* strains and feeding assays

*Drosophila* were obtained from the Bloomington Drosophila Stock Center (BDSC). All stocks were grown on standard culture medium at 22° C under a natural light-dark cycle. The GAL4 lines used in this study were driven by dopa decarboxylase (ddc). We used stocks with the UAS transgenes UAS-Flag-LRRK2-WT and UAS-LRRK2-G2019S [18].

For drug-feeding experiments, we added the appropriate concentrations of drug compounds dissolved in DMSO to a liquid mixture of 1.5% agar in grape juice supplemented with 3.2% inactivated yeast (w/v). In the liquid state, at 65° C, we plated 0.5 ml of this solution along one side of 4-ml plastic cuvette; the cuvette was held at an angle to ensure that the food mixture covered a large surface area. Drug compounds were added at two different concentrations, 10 or 20 mg/ml, in two separate cuvettes. Experiments with individual concentrations of each drug compound were repeated in triplicate. After the cuvettes cooled to 25° C, they were seeded with *Drosophila* eggs and then incubated at 25° C and 70% humidity until the larvae hatched. The newly hatched third instar larvae were collected with a brush and transferred to a new vial containing conventional medium for dendrite analysis.

### Immunostaining whole-mount dissected brains and cell counting

Cohorts of 8–10 adult flies (4 weeks old) per experimental group were used for immunostaining. Whole-mount dissected brains were subjected to fluorescence immunostaining with rabbit polyclonal anti-TH (TH; Pel-Freez). Secondary antibodies were Alexa Fluor 488 goat anti-mouse IgG and Alexa Fluor 568 goat anti-mouse IgG (Invitrogen). We scored the number of dopaminergic neurons under a confocal microscope (LSM710 inverted; Carl Zeiss).

### Transgenic LRRK2-WT and LRRK2-G2019S mice

We obtained transgenic *LRRK2* BAC-overexpressing human wild-type (FVB/N-Tg(LRRK2)1Cjli/J) and mutant *LRRK2-G2019S* mice (FVB/N-Tg(LRRK2*G2019S)1Cjli/J) from Jackson Laboratories. These mice were bred with non-transgenic FVB/N mice, keeping transgenic mice at the hemizygous state. The mutant mice were viable and fertile, and exhibited no gross morphological or behavioral abnormalities until the age of 12 months (data not shown), which is consistent with previous observations [44]. Animal experiments were approved by the ethics committee of National Taiwan University Hospital.

### Preparation of primary hippocampal and dopaminergic neurons

We prepared cultures of dissociated primary hippocampal or substantial nigral neurons from embryonic day 18 (E18) as described previously [45]. Briefly, on E18, the brains were removed and placed in Hank’s Balanced Salt Solution (HBSS, Gibco) on ice. Suspensions of isolated hippocampal or nigral neurons were prepared and used to seed six-well plates with 1 ml of plating medium, i.e., neurobasal medium with 0.5 mM α-glutamine (Gibco), 2% B27, and 1/100 penicillin/streptomycin (Invitrogen). After 4 days *in vitro* (DIV4), 10% of the media was replaced every 3–5 days.

### Immunofluorescence confocal microscopy

Primary hippocampal or nigral neurons were fixed with 4% paraformaldehyde (PFA) plus 4% sucrose for 10 minutes. The primary antibodies used in this study were anti-microtubule associated protein 2 (MAP2, Abcam) and anti-TH (Abcam). The secondary antibodies were anti-mouse Alexa-488, anti-rabbit Alexa568, and anti-chicken AMCA (Molecular Probes and Jackson Laboratories). Analyses and image acquisitions were performed using an Olympus Fluoview1000 confocal microscope.

### Stably transfected *LRRK2-G2019S* SH-SY5Y cells

Human neuroblastoma SH-SY5Y cells stably transfected with *LRRK2-G2019S* were a kind gift from Dr. Han Seok Ko [20]. We maintained all human neuroblastoma SH-SY5Y cells in DMEM plus 10% FBS. The SH-SY5Y cells were transfected with pcDNA3.1-FLAG-WT-LRRK2 or G2019S-LRRK2 using Lipofectamine Plus (Invitrogen) according to the manufacturer’s instructions. Two days later, selection was initiated using medium containing 700 μg/ml geneticin (Invitrogen). Individual clones were isolated and characterized by Western blot analysis with anti-FLAG antibody.

### Analysis of neurite outgrowth

Neurite lengths were manually quantified in differentiated SH-SY5Y cells stimulated by 24-h treatment with retinoic acid and primary hippocampal or dopaminergic neurons with individual genotypes treated with different concentrations of JMF3086 using Image J software (National Institutes of Health, Bethesda, Maryland, USA) as described previously [13]. Briefly, digital images of 10 fields of view (using a 10x objective lens) were taken of SH-SY5Y cells or primary neurons using an inverted light microscope (Leica) attached to an Infiniti X digital camera. Image J software was used to assess the mean neurite length from the total number of cells expressing neurites (Supplementary Figure 3G). A neurite was defined as a process extending from the cell body > 20 microns in length. Only cells with both their cell bodies and processes completely within the frame were analyzed. The mean neurite length per image was then averaged for the 10 fields of view to obtain the average neurite length per treatment.

### Western blot analysis

Proteins were separated by SDS-PAGE and then blotted onto a polyvinylidene difluoride (PVDF) membrane. Non-specific binding was blocked with Tris-buffered saline plus 5% milk powder and 0.2% Tween 20. The membranes were incubated with antibodies overnight at 4° C. We used the following primary antibodies: mouse anti-p44/42 MAPK (p-ERK, 1:5000; Cell Signaling, Danvers, MA, USA), rabbit anti-phospho-p44/42 ERK1/2 (Thr202/Tyr204, 1:2000; Cell Signaling), rabbit anti-Akt (1:2000, Cell Signaling), rabbit anti-phospho-Akt (Ser473, 1:2000; Cell Signaling), rabbit anti-Nrf (1:2000, GeneTex), mouse anti-caspase-3 (1:3000, Cell Signaling), rabbit anti-GSK3β (1:2000, Abcam), rabbit anti-phospho-GSK3β (pSer9, 1:2000; Abcam), mouse anti-Tau 1 (1:1000; EMD Millipore), mouse anti-phospho-paired helical filament-tau (pThr212/Ser214, AT100, 1:1000; Thermo Fisher Scientific), rabbit anti-β-actin (1:5000, GeneTex), rabbit monoclonal anti-LRRK2 ([MJFF2 (c41-2)], 1:1000; ab133474), rabbit monoclonal anti-phospho-LRRK2 (p-Ser935, 1:1000; ab133450), rabbit anti-MEK1/2 (1:1000; 9122S, Cell Signaling), rabbit anti-phospho-MEK1/2 (Ser217/221, 1:1000; 9154T, Cell Signaling), rabbit anti-HDAC6 (1:1000; 7558T, Cell Signaling), rabbit anti-phospho-HDAC6 (p-S22, 1:1000; ab61058, Cell Signaling), rabbit anti-caspase-3 (1:3000, Cell Signaling), and mouse anti-α-tubulin (1:5000, Sigma). The primary antibodies were recognized by either a horseradish peroxidase (HRP)-conjugated goat anti-rabbit secondary antibody (1:10,000; GeneTex) or an HRP-conjugated rabbit anti-mouse secondary antibody (1:10,000, GeneTex). Secondary antibodies were detected by enhanced chemiluminescence and the ECL Plus detection system (GE Healthcare). Resolved protein band sizes were determined using the precision protein dual color standard (Bio-Rad). For detection of phospho-Akt, phospho-GSK3β, and phospho-tau (AT100), we pre-treated the protein extracts with phosphatase inhibitors before Western blot analysis.

### Data analysis

Quantitative data were expressed as mean ± standard error of the mean (SEM). Significance (Bonferroni corrected) was assessed using the Student’s *t*-test for data with a normal distribution or a non-parametric *t*-test for data with a skewed distribution. We evaluated the effect of multiple factors using two-way ANOVA. *P*<0.05 was considered significant.

## Supplementary Material

Supplementary Figures
