# Supplementary material for: A dual inhibitor targeting HMG-CoA reductase and histone deacetylase mitigates neurite degeneration in LRRK2-G2019S parkinsonism
Source: Aging (Albany NY). 2020 Nov 24;12(24):25581–98. doi: 10.18632/aging.104165 (PMC7803522; doi:10.18632/aging.104165)
Supplement: Supplementary Figures [file aging-12-104165-s001.pdf]

SUPPLEMENTARY FIGURES

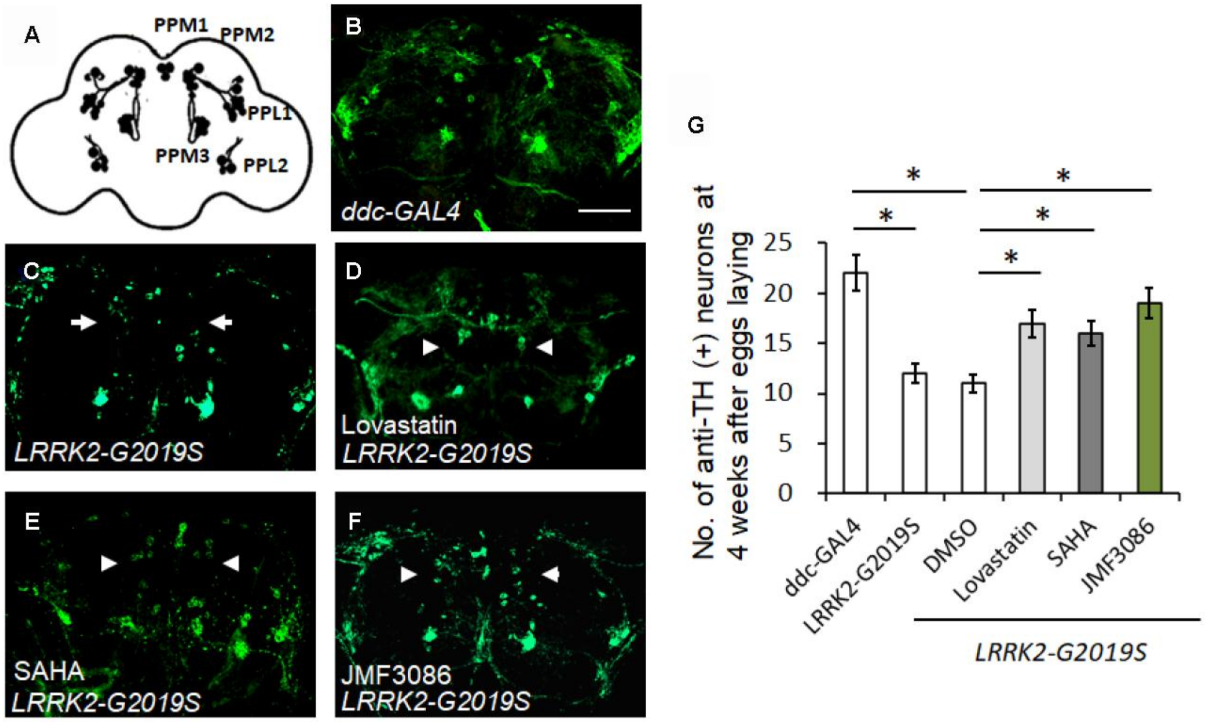

Supplementary Figure 1. Effects of lovastatin, SAHA, and JMF3086 at a feeding concentration of 20 mg/ml on the loss of tyrosine hydroxylase (TH)-positive cells in transgenic *LRRK2-G2019S* adult flies. (**A**–**F**) Whole-mount adult brains from 4-week-old *ddc-GAL4* control and transgenic *LRRK2-G2019S* flies were stained with anti-TH antibodies (*green*) to label individual dopaminergic neuronal clusters in 4-week-old flies. Images are representative of (**A**) Schematic representation of the distribution of dopaminergic neurons in the *Drosophila* adult brain. Dopaminergic neurons are grouped in small clusters arranged with bilateral symmetry. PPM, protocerebral posterior medial; PPL, protocerebral posterior lateral. Protocerebral posterior medial and lateral neuronal clusters are shown before treatment in *ddc-GAL4* control (**B**) and transgenic *LRRK2-G2019S* flies (**C**) after treatment with (**C**) solvent DMSO, (**D**) 20 mg/ml lovastatin, (**E**) 20 mg/ml SAHA, and (**F**) 20 mg/ml JMF3086. (**G**) Quantification of TH-positive neurons in protocerebral posterior medial and lateral neuronal clusters in *ddc-GAL4* control and transgenic *LRRK2-G2019S* brains from 4-week-old flies treated with different drug compounds (For PPM1/2 and PPL1/2, *ddc-GAL4* vs. *LRRK2-G2019S* without compound treatment was 22.18±2.23 vs 12.35±1.59, *P*=0.02; *ddc-GAL4* vs. *LRRK2-G2019S* with DMSO solvent was 22.18±2.23 vs 11.25±1.78, *P*=0.02; *LRRK2-G2019S* with DMSO solvent vs. *LRRK2-G2019S* with 0.5 μM lovastatin was 11.25±1.78 vs 17.26±2.59, *P*=0.03; *LRRK2-G2019S* with DMSO solvent vs. *LRRK2-G2019S* with 0.5 μM SAHA was 11.25±1.78 vs 16.12±2.31, *P*=0.03; *LRRK2-G2019S* with DMSO solvent vs. *LRRK2-G2019S* with 0.5 μM JMF3086 was 11.25±1.78 vs 19.23±2.56, *P*=0.02; by 1-way ANOVA). Scale bar, 20 μm. Data represent mean ± SEM. *$P<0.05$, **$P<0.01$.

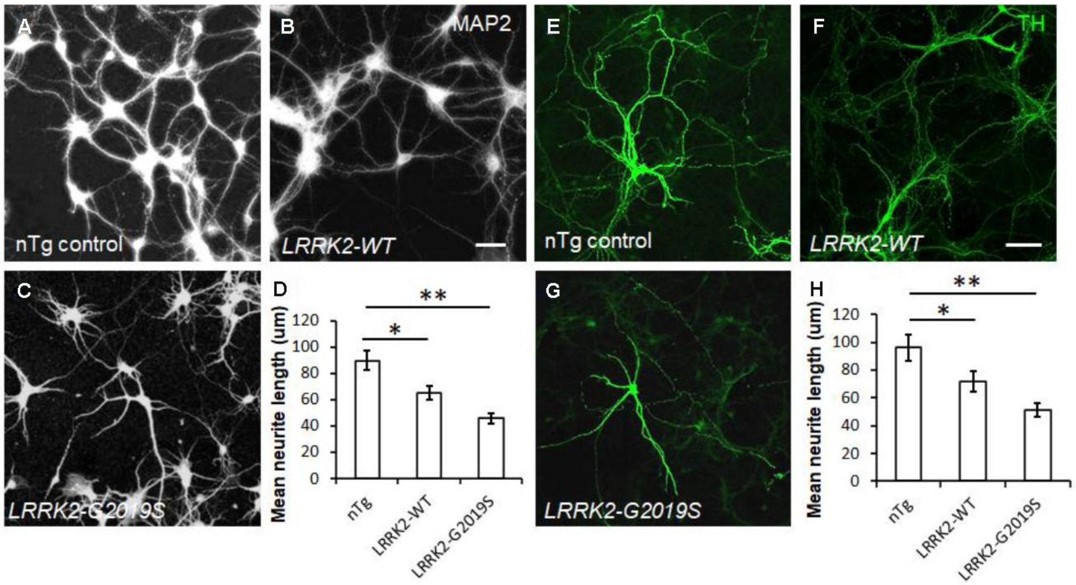

Supplementary Figure 2. Neurite arborization phenotypes in primary hippocampal and primary TH (+) nigral neurons from transgenic LRRK2 wild-type and LRRK2-G2019S mice. Representative images show cultured primary hippocampal neurons (DIV 14) from non-transgenic (nTg) littermate controls (**A**), transgenic *LRRK2* wild-type (*LRRK2-WT*) (**B**), and transgenic *LRRK2-G2019S* (**C**) pups. Neurites were stained with anti-MAP2 antibodies. Scale bar, 100 μm. (**E**–**G**) Representative images show cultured primary nigral TH-positive neurons (DIV 14) from nTg littermate controls (**E**), *LRRK2* wild-type (**F**), and *LRRK2-G2019S* (**G**) pups. Neurites were stained with anti-TH antibodies. Scale bar, 100 μm. (**D**, **H**) Quantitative analyses of mean total neurite lengths for the primary hippocampal neurons described in (**A**–**D**) and for the primary nigral TH-positive neurons described in (**E**–**H**). We analyzed 50–100 neurons from each genotype of primary hippocampal neurons, and 20–30 for each genotype of primary nigral TH-positive neurons. For primary hippocampal neurons, nTg littermate controls: 92.6±9.8 μm, *LRRK2-WT*: 65.3±8.5 μm, *LRRK2-G2019S*: 43.7±6.3 μm; 1-way ANOVA *P*=0.03 for nTg littermate controls vs. *LRRK2-WT*; *P*=0.007 for *UQCRC1 LRRK2-WT* vs. *LRRK2-G2019S*. For primary TH (+) nigral neurons, nTg littermate controls: 98.1±12.3 μm, *LRRK2-WT*: 77.5±8.2 μm, *LRRK2-G2019S*: 58.9±8.6 μm; 1-way ANOVA *P*=0.05 for nTg littermate controls vs. *LRRK2-WT*; *P*=0.009 for *UQCRC1 LRRK2-WT* vs. *LRRK2-G2019S*. Data represent mean ± SEM. *$P<0.05$, **$P<0.01$.

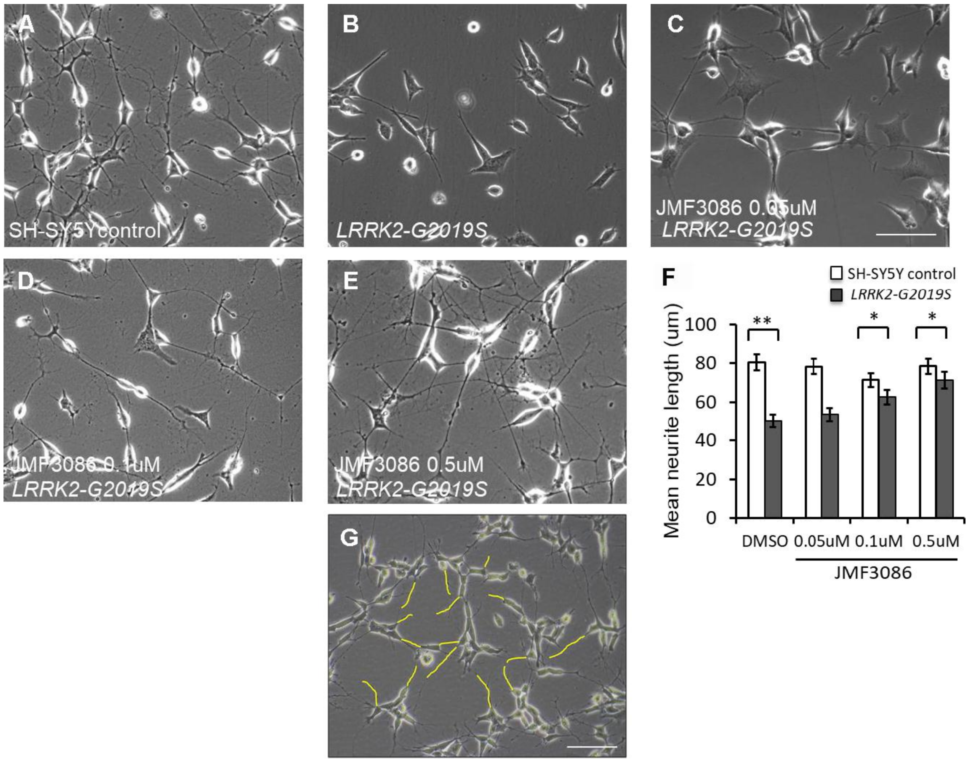

Supplementary Figure 3. JMF3086 mitigates neurite degeneration in SH-SY5Y cells stably transfected with *LRRK2-G2019S*. (**A**, **B**) SH-SY5Y cell morphology observed with light microscopy (Nikon Eclipse, 80i; 10× magnification). Compared to control neurons (**A**), *LRRK2-G2019S* neurons showed markedly decreased neurite branching and length (**B**). (**C**–**E**) *LRRK2-G2019S* SH-SY5Y cells were treated with 0.05 µM (**C**), 0.1 µM (**D**), or 0.5 µM JMF3086 (**E**). (**F**) Quantitative analyses of mean total neurite lengths described in A–E. (**G**) SH-SY5Y neurite outgrowth was traced by yellow lines and the mean neurite length measured for total number of cells in each field of view was measured. Scale bar, 100µm. We analyzed 50–100 neurons for each genotype or treatment condition. Scale bar, 100µm. Data represent mean ± SEM; **P*<0.05, ***P*<0.01.
